# Cortical thickness abnormalities in patients with first episode psychosis: a meta-analysis of psychoradiologic studies and replication in an independent sample

**DOI:** 10.1093/psyrad/kkab015

**Published:** 2021-12-15

**Authors:** Keren Wen, Youjin Zhao, Qiyong Gong, Ziyu Zhu, Qian Li, Nanfang Pan, Shiqin Fu, Joaquim Radua, Eduard Vieta, Poornima Kumar, Graham J Kemp, Bharat B Biswal

**Affiliations:** Huaxi MR Research Center (HMRRC), Department of Radiology, West China Hospital of Sichuan University, Chengdu 610041, Sichuan, China; Huaxi MR Research Center (HMRRC), Department of Radiology, West China Hospital of Sichuan University, Chengdu 610041, Sichuan, China; Research Unit of Psychoradiology, Chinese Academy of Medical Sciences, Chengdu 610041, Sichuan, China; Huaxi MR Research Center (HMRRC), Department of Radiology, West China Hospital of Sichuan University, Chengdu 610041, Sichuan, China; Research Unit of Psychoradiology, Chinese Academy of Medical Sciences, Chengdu 610041, Sichuan, China; Functional and Molecular Imaging Key Laboratory of Sichuan Province, Chengdu 610041, Sichuan, China; Huaxi MR Research Center (HMRRC), Department of Radiology, West China Hospital of Sichuan University, Chengdu 610041, Sichuan, China; Huaxi MR Research Center (HMRRC), Department of Radiology, West China Hospital of Sichuan University, Chengdu 610041, Sichuan, China; Huaxi MR Research Center (HMRRC), Department of Radiology, West China Hospital of Sichuan University, Chengdu 610041, Sichuan, China; Huaxi MR Research Center (HMRRC), Department of Radiology, West China Hospital of Sichuan University, Chengdu 610041, Sichuan, China; Institut d'Investigacions Biomèdiques August Pi i Sunyer (IDIBAPS), Mental Health Research Networking Center (CIBERSAM), Barcelona 08036, Catalonia, Spain; Centre for Psychiatric Research and Education, Department of Clinical Neuroscience, Karolinska Institutet, Solna 171-77, Stockholm, Sweden; Department of Psychosis Studies, Institute of Psychiatry, Psychology and Neuroscience, King's College London, London WC2R 2LS, UK; Institut d'Investigacions Biomèdiques August Pi i Sunyer (IDIBAPS), Mental Health Research Networking Center (CIBERSAM), Barcelona 08036, Catalonia, Spain; Barcelona Bipolar Disorders and Depressive Unit, Hospital Clinic, Institute of Neurosciences, University of Barcelona, Barcelona 08036, Catalonia, Spain; Center for Depression, Anxiety and Stress Research, McLean Hospital, Belmont 02478, MA, USA; Department of Psychiatry, Harvard Medical School, Boston 02115, MA, USA; Liverpool Magnetic Resonance Imaging Centre (LiMRIC) and Institute of Life Course and Medical Sciences, University of Liverpool, Liverpool L69 3GE, UK; Department of Biomedical Engineering, New Jersey Institute of Technology, Newark 07102, NJ, USA; The Clinical Hospital of Chengdu Brain Science Institute, MOE Key Laboratory for Neuroinformation, University of Electronic Science and Technology of China, Chengdu 610054, Sichuan, China

**Keywords:** psychoradiology, first episode psychosis, early psychosis, cortical thickness, meta-analysis, seed-based d mapping

## Abstract

**Background:**

Abnormalities of cortical thickness (CTh) in patients with their first episode psychosis (FEP) have been frequently reported, but findings are inconsistent.

**Objective:**

To define the most consistent CTh changes in patients with FEP by meta-analysis of published whole-brain studies.

**Methods:**

The meta-analysis used seed-based d mapping (SDM) software to obtain the most prominent regional CTh changes in FEP, and meta-regression analyses to explore the effects of demographics and clinical characteristics. The meta-analysis results were verified in an independent sample of 142 FEP patients and 142 age- and sex-matched healthy controls (HCs), using both a vertex-wise and a region of interest analysis, with multiple comparisons correction.

**Results:**

The meta-analysis identified lower CTh in the right middle temporal cortex (MTC) extending to superior temporal cortex (STC), insula, and anterior cingulate cortex (ACC) in FEP compared with HCs. No significant correlations were identified between CTh alterations and demographic or clinical variables. These results were replicated in the independent dataset analysis.

**Conclusion:**

This study identifies a robust pattern of cortical abnormalities in FEP and extends understanding of gray matter abnormalities and pathological mechanisms in FEP.

## Introduction

First episode psychosis (FEP) refers to the initial stage of psychosis (Breitborde *et al*., 2009), usually characterized by distortions in cognition, behavior, and physical function (Chudleigh *et al*., 2011; Grant *et al*., 2001). FEP is common in young adults (Reed, 2008) and has high rates of disability and mortality (Simon *et al*., 2018). With the development of psychoradiology (Gong, 2020; Lui *et al*., 2016), an abundance of structural magnetic resonance imaging (MRI) studies have provided key information for neurostructural changes in FEP (Li *et al*., 2021). Applying surface-based morphometric methods allows quantification of cortical thickness (CTh) (Fishcl and Dale, 2000). CTh reflects the density, arrangement, and size of neurons, neuroglia, and nerve fibers (Narr *et al*., 2005), and is only minimally affected by partial volume effects (Winkler *et al*., 2010). CTh abnormalities reflect regional disease-specific effects: cortical thinning can follow a loss of dendrites and dendritic spines or alterations during myelination, while neuroinflammation or other factors can increase CTh (Hutton *et al*., 2008; Narr *et al*., 2005; Sowell *et al*., 2004; Winkler *et al*., 2010).

Recent studies in FEP have reported extensive CTh alterations in salience processing, cognitive, and emotional areas involving the default mode network (DMN) and the salience network (SN). However, reported results are inconsistent: compared with healthy controls (HCs), lower CTh has been reported in the superior (Ansell *et al*., 2015; Haring *et al*., 2016; Plitman *et al*., 2016), middle (Ansell *et al*., 2015; Buchy *et al*., 2017; Plitman *et al*., 2016), and inferior (Plitman *et al*., 2016; Qiu *et al*., 2013) frontal gyri, superior (Plitman *et al*., 2016; Schultz *et al*., 2010) and inferior parietal (Ansell *et al*., 2015; Bodnar *et al*., 2014) gyri; superior (Scanlon *et al*., 2014; Schultz *et al*., 2010; Song *et al*., 2015) and middle temporal gyri (Kim *et al*., 2012; Qiu *et al*., 2013; Scanlon *et al*., 2014); insula (Song *et al*., 2015); and cingulate gyri (Bodnar *et al*., 2014; Schultz *et al*., 2010). Higher CTh has been reported in the middle temporal gyrus (Qiu *et al*., 2013), temporal pole (Haring *et al*., 2016; Xiao *et al*., 2015), precentral (Ansell *et al*., 2015; Haring *et al*., 2016), and postcentral gyri (Ansell *et al*., 2015), and bilateral superior, middle, and inferior occipital gyri (Ansell *et al*., 2015; Dukart *et al*., 2017). Some studies report no significant differences of CTh (Lesh *et al*., 2015; Lin *et al*., 2019; Reniers *et al*., 2015). Causes of these inconsistencies may include differences in sample characteristics (e.g. patient demographics, symptom severity, illness duration, medication status, and sample size), MRI data acquisition methods, and processing protocols. A meta-analysis is urgently needed to resolve these apparent contradictions. For brain imaging studies, coordinate-based meta-analysis can synthesize results from different studies in an unbiased way, yielding a robust picture of cortical alterations in disease (Quah and Cockerham, 2017).

Therefore, we aimed to conduct a meta-analysis of whole brain CTh studies in patients with FEP by using seed-based d mapping (SDM) software. We used a mask specifically designed for whole-brain CTh, which minimizes the effect of subcortical gray matter (Li *et al*., 2020). We performed meta-regression analyses to explore the effects of demographics and clinical characteristics on CTh. We also investigated whether the CTh findings in our meta-analysis could be replicated in an independent FEP sample: we used both a whole-brain vertex-wise analysis, and also (to directly compare cerebral areas identified in the meta-analysis) a region of interest (ROI)-based analysis. As previous studies report widespread disruptions of CTh in the DMN and SN, we hypothesized that, compared with HCs, FEP patients would show CTh alterations in DMN and SN involving core regions for cognitive and emotional function, possibly associated with demographics and clinical characteristics.

## Materials and Methods

### Meta-analysis of cortical thickness studies

#### Study selection

A comprehensive strategy was executed in September 2020 to search for pertinent literature in Web of Science, PubMed, Embase, and Science Direct databases. The search terms included: (“first-episode psychosis” OR “first-episode schizophrenia” OR “first-episode bipolar disorder” OR “first-episode depression” OR “early psychosis” OR “early schizophrenia” OR “early bipolar disorder” OR “early depression” OR “drug-naïve psychosis” OR “drug-naïve schizophrenia” OR “drug-naïve bipolar disorder” OR “drug-naïve depression”) AND (“cortical thickness”). In addition, we manually checked the references of these studies to identify further studies for inclusion. Studies meeting the following criteria were included: (i) studies published in English in a peer-reviewed journal; (ii) studies that recruited individuals who met the diagnostic criteria of FEP and healthy controls based on the cross-diagnostic approach (Fusar-Poli and Meyer-Lindenberg, 2013a; Fusar-Poli and Meyer-Lindenberg, 2013b), where FEP was defined as several mental illnesses characterized by psychotic symptoms including schizophrenia spectrum psychoses (schizophrenia, schizoaffective, schizophreniform) and affective psychoses (bipolar psychosis and psychotic depression); (iii) studies that used whole-brain CTh analysis methods; and (iv) studies that documented Montreal Neurological Institute (MNI) or Talairach (TAL) coordinates of peak CTh alterations, or reported no significant findings. Reviews and theoretical papers were excluded. For studies using overlapping samples, we only included the study with largest samples. For longitudinal studies, we only included baseline data to avoid bias toward the effect of interventions or illness progression. For studies containing multiple independent patient samples, results were considered as separate datasets. Corresponding authors were contacted if the peak coordinates of effects were not available in whole-brain CTh studies. Two authors independently conducted the literature search, and any inconsistencies were discussed to reach an agreement. The flow of literature selection is summarized in Supplementary Fig. S1.

#### Quality assessment and data recording

Primary studies included in meta-analysis were assessed using a modified version of Newcastle-Ottawa Scale (NOS) that scores potential risk of bias for case-control studies in terms of six aspects of study quality (Keramatian *et al*., 2021; Wells *et al*., 2000). The details are given in Supplementary Table S1. The recorded information for each included dataset consisted of clinical characteristics (sample numbers, gender, mean age of participants, mean age at onset, mean illness duration, mean duration of untreated psychosis (DUP), mean symptom severity, medication status, diagnosis), imaging characteristics (scanner manufacturer and model, field strength, sequence name, spatial resolution, normalization template, repetition time/echo time, data processing software, analytic model, method to correct whole-brain results for multiple comparisons, and statistical threshold of the main findings), and the main CTh alterations. Peak coordinates and corresponding *t, P*, or *z* values were also recorded for SDM calculations. If only *P* or *z* values were available, these were converted into *t* values (O'Neill *et al*., 2019).

#### Protocol for meta-analysis

First, we performed a pooled meta-analysis with all included studies. Then subgroup meta-analyses of studies using 3.0 T and 1.5 T MRI scanners were performed to investigate the possible effects of field strength. SDM (www.sdmproject.com) software (Kimmel *et al*., 2016; Zhang *et al*., 2016) was used to execute the meta-analysis. The details have been presented in detail elsewhere (Radua *et al*., 2011; Radua and Mataix-Cols, 2009; Radua *et al*., 2014b), and we give only a brief summary here. First, reported peak coordinates and *t* values of significant differences between FEP patients and HCs were used to recreate an effect-size signed map for each study. Importantly, applying the same threshold throughout the whole brain in each included study avoided biases toward brain regions with liberal thresholds (Radua *et al*., 2011; Radua *et al*., 2014b). Second, we chose a specially constructed mask (the details of which can be found in (Li *et al*., 2020)) to restrict the analysis to the cortex. Third, the mean map was derived using a traditional random-effects meta-analytic methods, with both negative and positive changes presented in the same map (Radua and Mataix-Cols, 2009). We used SDM's default threshold for analyses (voxel   *P<* 0.005, peak height *z* > 1, cluster extent  =  10 voxels).

#### Jackknife, heterogeneity and publication bias analysis

Replicability was assessed by jackknife sensitivity analysis. The main statistical analysis was repeated *N* times (*N* = number of datasets in the meta-analysis) by discarding one dataset at a time to determine whether the results remained significant. We estimated the statistical (between-studies) heterogeneity of individual clusters using *q* statistics (*χ*^2^ distribution converted to *z* values) and tested for heterogeneity of findings with a permutation approach. The possibility of publication bias for regions showing altered CTh was examined using Egger tests.

#### Meta-regression analysis

Meta-regression analyses were conducted to examine the effects of age, illness duration (mean values), gender (percentage of females), and medication status (percentage of medicated participants) on CTh. Meta-regression analysis of age at onset and symptom severity was not possible, being reported in fewer than nine studies (Radua and Mataix-Cols, 2009). In accordance with our previous meta-analysis, the probability threshold was reduced to 0.0005 to minimize detection of spurious relationships (Radua *et al*., 2011; Radua and Mataix-Cols, 2009), we required abnormalities to be detected both in the slope and in one of the extremes of the regressor, any regions not detected in the main analyses were discarded, and regression plots were visually inspected to discard findings driven by too few studies (Radua and Mataix-Cols, 2009).

### Validation of meta-analysis results in an independent sample of patients and controls

To verify the results from the meta-analysis, a study was conducted using both whole-brain vertex-wise and ROI-based analyses to compare CTh between an independent group of FEP patients and controls.

#### Participants and MRI data acquisition

We recruited 142 patients with FEP and 142 age- and sex-matched HCs, and acquired high-resolution T1-weighted images. The severity of clinical symptoms and psychosocial functioning were evaluated using the Positive and Negative Syndrome Scale (PANSS) (Kay *et al*., 1987) and the Global Assessment Function (GAF) scale (Morosini *et al*., 2000). The inclusion and exclusion criteria and MRI acquisition parameters are given in the Supplementary Methods. This study was approved by the ethics committee of Sichuan University, China. Written informed consent was obtained from each participant.

#### Image processing

The processing of structural images was performed in FreeSurfer software package 6.0.0 (Fischl, 2012) (http://surfer.nmr.mgh.harvard.edu) using the following steps: (i) head motion correction, (ii) skull stripping, (iii) transformation to the Talairach space, (iv) segmentation of gray/white matter, (v) surface inflation and registration to a spherical atlas, (vi) CTh calculation as the shortest distance between the gray–white interface and the pial interface (Poppa and Bechara, 2018), and (vii) surface-map smoothing using a Gaussian kernel with a full-width at half-maximum of 20 mm.

#### Statistical analysis

As a preliminary analysis, Kolmogorov–Smirnov (KS) tests, Shapiro–Wilk (SW) tests, and visual assessment of the histogram were comprehensively used to analyze the normality of clinical variables (Ghasemi and Zahediasl, 2012). For normally distributed continuous variables, two-sample *t*-tests were used. For nonnormally distributed continuous variables, Mann–Whitney tests were used. For gender distribution, the chi-square test was used. All tests were two-tailed, and statistical significance was considered at *P* < 0.05.

In the independent sample, whole-brain vertex-wise analysis assessed the group differences of CTh using a general linear model (GLM) based on QDEC (query, design, estimate, contrast) in FreeSurfer. Monte Carlo simulation controlled for multiple comparisons (cluster-wise corrected *P* < 0.05). Correlations between CTh and age, GAF, and PANSS scores were separately examined for each vertex in the FEP group. Statistical significance of correlations was set at *P* < 0.05 after correction for multiple comparisons using the criteria of false discovery rate (FDR).

Results were also confirmed using an ROI method. First, we created ROI masks based on the meta-analysis results (Supplementary Fig. S5). Next, we extracted the average CTh of each ROI for each participant using the FreeSurfer command mris_anatomical_stats. Before intergroup comparisons and correlation analysis, the normality of CTh values of each ROI was analyzed using KS tests, SW tests, and visual assessment of the histograms. Then, the CTh values of each ROI were compared between FEP patients and HCs using two-sample *t*-tests for normally distributed variables and Mann–Whitney tests for nonnormally distributed data (*P* < 0.05, FDR corrected). Finally, correlations (Pearson correlation for normally distributed data and Spearman rank correlation for nonnormally distributed data) (Schober *et al*., 2018) between CTh and age, GAF and PANSS scores were separately examined for each ROI in the FEP group (*P* < 0.05, FDR corrected).

## Results

### The characteristics of the included studies

The meta-analysis incorporated 10 studies, with a total sample of 624 patients with FEP (213 females (34%), mean age 24.2 years), and 505 healthy controls (193 females (38%), mean age 24.5 years). Three of the 10 studies recruited two subgroups depending on the medication type (Ansell *et al*., 2015), medication status (Lesh *et al*., 2015) and history of cannabis use (Rais *et al*., 2010): Ansell *et al*. separated 52 FEP patients into two medication-type datasets: first-generation antipsychotics (FGA) and second-generation antipsychotics (SGA) included here as two datasets (Ansell *et al*., 2015). Lesh *et al*. studied two subgroups, unmedicated and medicated. We included only the former, as peak coordinates of CTh alterations were not available for the latter (Lesh *et al*., 2015). Rais *et al*. included two subgroups, cannabis-using and non-using (Rais *et al*., 2010); to reduce confounding factors we only included the latter. Thus, there were 11 datasets from 10 studies in the meta-analysis, and no significant differences were found in mean age (*t* = −0.298, *P* = 0.783) and gender (*χ*² = 1.745, *P* = 0.187) between FEP patients and HCs. The clinical characteristics of included datasets are listed in Table 1, and the imaging characteristics and main CTh alterations are listed in Supplementary Table S2.

**Table 1:** The clinical characteristics of the 11 datasets included.

	Numbers (female)		Age (y)							
Paper	FEP	HC	FEP	HC	Age at onset (y)	Illness duration	Symptom severity	Medication status ^d^	Diagnosis: numbers	Main findings
Ansell *et al*., 2015 FGA ^a^	25 (8)	28 (11)	21.9	21.1	NA	42 d	PANSS: 22.4 (pos), 22.0 (neg), 41.6 (gen)	25/161/39	SCZ: 15; depression disorders and BD: 10	Cortical thinning: right rostral, middle frontal and superior frontal gyri
Ansell *et al*., 2015 SGA ^a^	27 (9)	28 (11)	21.9	21.1	NA	93 d	PANSS: 22.4 (pos), 21.9 (neg), 44.3 (gen)	27/252/165	SCZ: 12; depression disorders and BD: 15	Cortical thinning: right inferior parietal, superior frontal, and fusiform gyri; cortical thickening: left precentral and right postcentral gyri
Buchy *et al*., 2018	130 (37)	52 (15)	24.1	24.3	NA	5.8 y	SAPS: 4.0 (total pos), 1.5 (delusions); SANS: 8.6; Self-reflectiveness: 12.4; Self-certainty: 7.9; CDSS: 2.3	130/793/NA	SCZ: 78; SAD: 13; SCZ-F: 2; BD: 14; MDD with psychotic features: 1; DD: 3; psychosis NOS: 11;	No significant CTh alteration by whole-brain vertex-wise method
Dukart *et al*., 2017	59 (17)	26 (14)	26.4	27.7	NA	NA	SANS: 18.0; BPRS: 49.7	28/216/NA	NA	Cortical thickening: left superior, middle and inferior occipital gyri, occipital pole, fusiform gyri, and cerebellum exterior, right superior, middle and inferior occipital gyri, occipital pole, cerebellum exterior, fusiform and lingual gyrus, calcarine cortex
Gutierrez *et al*., 2010	37 (12)	38 (16)	26.8	25.0	NA	10.4 m	WMS: 5.5; Planning: 7.2; WMM: 33.9; RAVLT: 38	37/NA/80	SCZ: 34; BD: 1; depressed subtypes: 2	No significant CTh alteration by whole-brain vertex-wise method
Haukvik *et al*., 2016	79 (27)	82 (28)	27.6	29.3	23.8	123 w	PANSS: 14.9 (pos), 14.0 (neg), 31.7 (gen); CDSS: 6.2; GAF: 43 (symptoms), 44 (function)	NA	SCZ: 37; SCZ-F: 2; SAD: 5; BD: 18; psychotic depression: 5; paranoid psychosis: 2; psychosis NOS: 10	No significant CTh alteration by whole-brain vertex-wise method
Lesh *et al*., 2015^b^	22 (3)	37 (10)	20.2	19.7	NA	210 d	SAPS: 7.5; SANS: 8.6; BPRS: 43.1; GAF: 43.5	0/0/0	SCZ/SAD/SCZ-F: 22	No significant CTh alteration in unmedicated group compared with HC
Lin, *et al*., 2019	145 (76)	147 (76)	24.5	25.9	23.6	11 m	PANSS: 25.2 (pos), 19.9 (neg), 48.1 (gen); GAF:29.5	0/0/0	SCZ/SCZ-F: 145	No significant CTh alteration by whole-brain vertex-wise method
Rais *et al*., 2010^c^	32 (6)	31 (6)	23.3	24.7	22.5	351 d	PANSS:18.8 (pos), 19.3 (neg)	18/NA/77	SCZ: 32	No significant CTh alteration in non-cannabis-using group compared with HC
Reniers *et al*., 2015	22 (4)	22 (4)	20.6	22.5	NA	3 w	SANS: 19.2; BPRS: 5.09 (pos), 9.09 (hostility and suspiciousness)	14/200/5	SCZ: 1; SAD: 3; SCZ-F: 9; SIPD: 2; psychosis NOS: 2; DD: 1; MDD: 2; bipolar unspecified: 1; BD: 1	No significant CTh alteration by whole-brain vertex-wise method
Scalon *et al*., 2014	46 (14)	46 (13)	28.4	28.6	NA	14 m	PANSS:17 (pos), 15 (neg), 33 (gen); GAF: 51	39/224/18	SCZ: 15; SAD: 4; SCZ-F: 5; DD: 3; mania: 9; psychotic depression: 6; psychosis NOS: 4	Cortical thinning: right superior temporal gyrus and sulcus, extending into middle temporal gyrus

a A study with two datasets; patients receiving first-generation and second-generation antipsychotics, respectively.

b A study with two datasets; only the nonmedication dataset is included because the peak effect coordinates of CTh alterations in the medication dataset were not available.

c This is from a study containing two datasets; only the dataset from patients who used no illicit drugs is included.

d Medicated number/chlorpromazine equivalent (mg)/treatment duration (days).

Abbreviations: BD, Bipolar disorder; BPRS, Brief Psychiatric Rating Scaled; d, day; CDSS, Calgary Depression Scale for Schizophrenia; DD, delusional disorder; DUP, duration of untreated psychosis; FGA, first-generation antipsychotics; GAF, Global Assessment of Functioning; gen, general; HARS, Hamilton Anxiety Rating Scale; MDD, major depressive disorder; m, month; NA, not applicable; neg, negative; NOS, not otherwise specified; PANSS, Positive And Negative Syndrome Scale; pos, positive; RAVLT, Rey Auditory Verbal Learning Test; SAD, schizoaffective; SANS, Scale for the Assessment of Negative Symptoms; SAPS, Schedule for the Assessment of Positive Symptoms; SGA, second-generation antipsychotics; SCZ, Schizophrenia; SCZ-F, Schizophreniform; SIPD, substance-induced psychotic disorder; w, week; WMM, working memory manipulation; WMS, working memory span; y, year; YMRS, Young Mania Rating Scale.

### Assessment of risk of bias

The potential risk of bias of the studies is described in Supplementary Figs S2 and S3. Four (40%) of the 10 studies included in the meta-analysis received a maximum score of 6 on the modified version of the NOS, five studies (50%) received a score of 5, and one study (10%) received a score of 4. All 10 studies are relatively low risk or with some concerns about bias, mostly because of no description about the selection of controls.

### Results of the pooled meta-analysis

**Figure 1:**
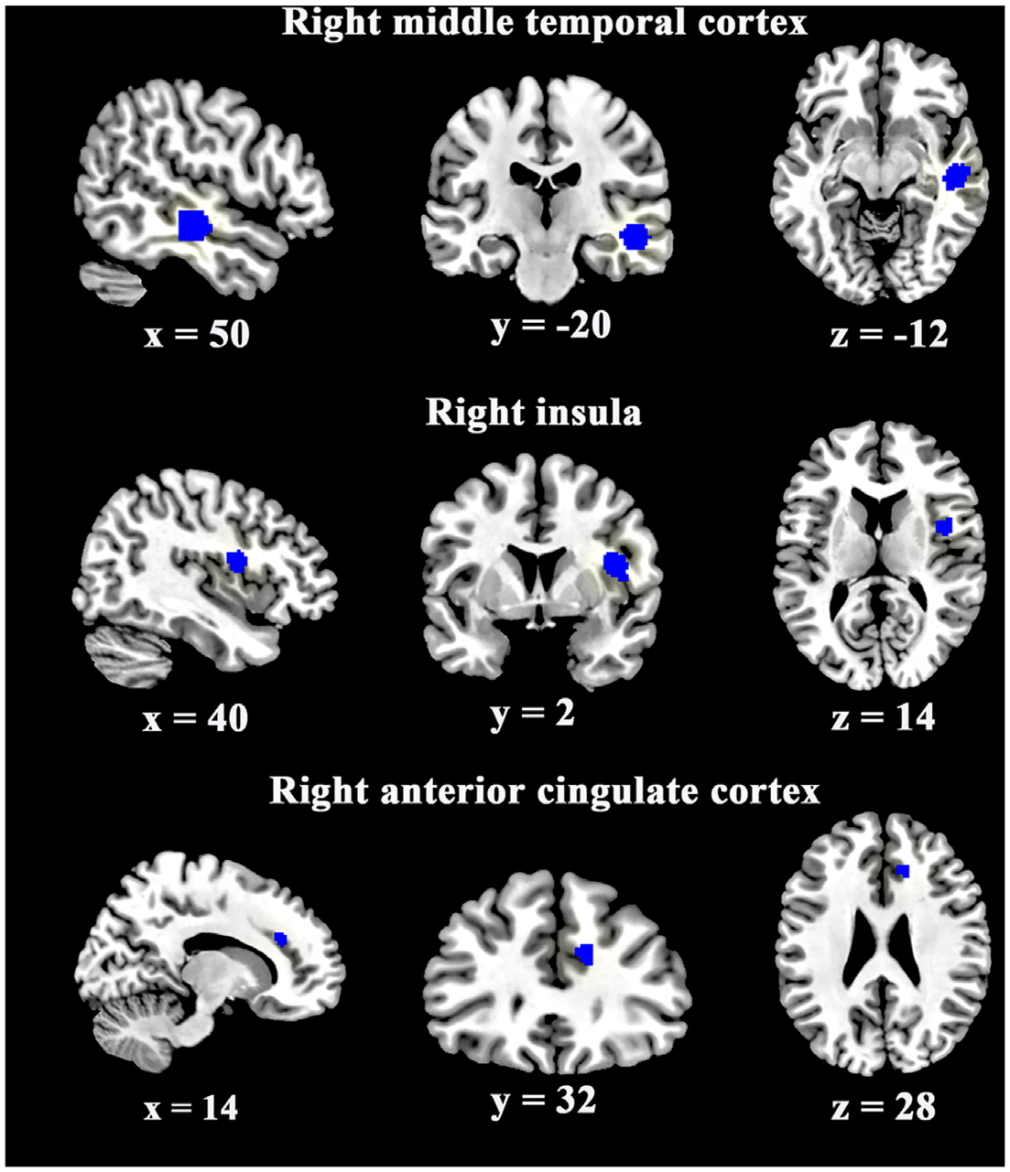
The areas and corresponding Montreal Neurological Institute (MNI) coordinates of decreased (blue) cortical thickness in participants with FEP compared with healthy controls in the pooled meta-analysis.

Compared with HCs, FEP patients showed lower CTh in the right middle temporal cortex (MTC) extending to superior temporal cortex (STC), insula, and anterior cingulate cortex (ACC) (Table 2 and Fig. 1).

**Table 2:** Three regions with decreased cortical thickness in patients with FEP compared to healthy controls.

	MNI coordinate							Effect size	
Brain regions	*x*	*y*	*Z*	SDM (*z* score)	*P*, uncorrected	Voxels (*n*)	Cluster breakdown (voxels, *n*)	Estimate	Variance
Right middle temporal cortex	50	−20	−12	−1.023	0.00 126	319	Right middle temporal cortex, BA 20, 21, 22, 48 (193)	−0.093	0.008
							Right superior temporal cortex, BA 21, 22, 48 (126)		
Right insular cortex	40	2	14	−1.136	0.00 051	161	Right insula cortex, BA 48 (96)	−0.143	0.016
							Right Rolandic operculum, BA 48 (65)		
Right anterior cingulate cortex	14	32	28	−1.001	0.00 175	53	Right anterior cingulate/paracingulate cortex, BA 32 (53)	−0.101	0.010

Abbreviations: BA, Brodmann area; MNI, Montreal Neurological Institute.

### Jackknife, heterogeneity, and publication bias

In whole-brain jackknife sensitivity analysis, lower CTh in the right MTC, insula, and ACC was highly replicable, being statistically significant in all but one combination (Supplementary Table S3). No statistically significant heterogeneity was detected. No statistically significant publication bias (Supplementary Fig. S4) was revealed in the right MTC (*P* = 0.662), insula (*P* = 0.188), and ACC (*P* = 0.243).

### Subgroup meta-analysis

For the eight datasets that used a 1.5 T scanner, FEP patients showed lower CTh in the right MTC extending to STC, insula, ACC, and inferior frontal cortex, and higher CTh in the left precentral cortex than HCs (Supplementary Table S4). For the three datasets that used a 3.0 T scanner, there were no significant differences between the two groups.

### Meta-regression analysis

Meta-regression analysis revealed no statistically significant correlation between CTh and age, gender, illness duration, or medication status (Supplementary Table S5).

### Validation of meta-analysis results in an independent sample

**Figure 2:**
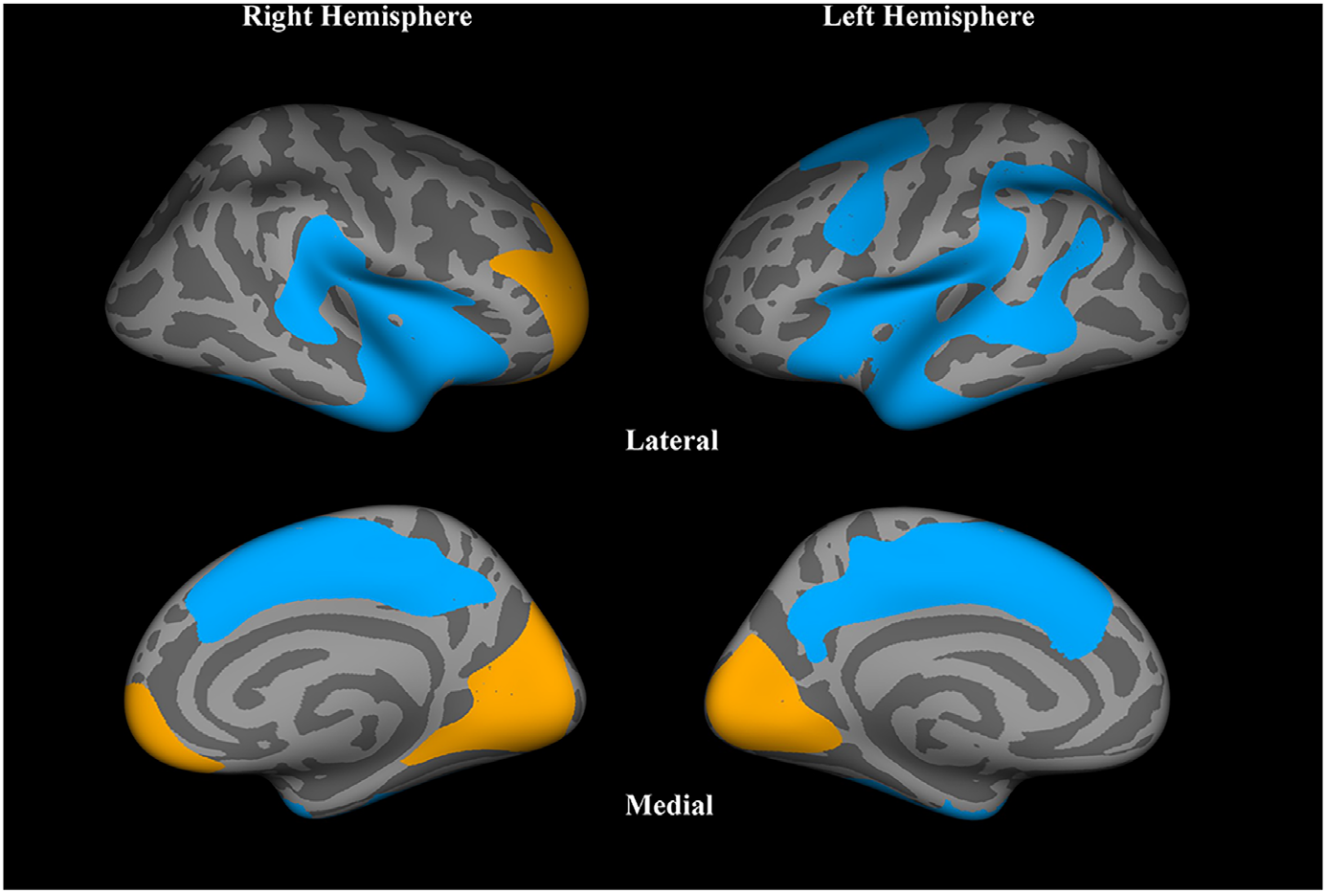
The areas showing lower (blue) and higher (orange) cortical thickness in participants with FEP compared with healthy control participants in the independent sample analysis.

The studied FEP patients and HCs did not significantly differ in age or gender. Table 3 summarizes their clinical and demographic characteristics. In the whole-brain analysis, FEP patients compared to HC showed significantly lower CTh in the bilateral STC (extending to MTC, temporal pole, insula, and fusiform) and superior frontal cortex (extending to anterior and posterior cingulate cortex), and higher CTh in the bilateral lingual, cuneus gyri, and right prefrontal cortex (Table 4 and Fig. 2). In the ROI analysis, FEP patients showed significantly lower CTh in the MTC, insula, and ACC (Supplementary Table S6). There was no significant association between CTh and clinical characteristics in either whole-brain or ROI analyses (Supplementary Table S7).

**Table 3:** Characteristics of the independent sample of patients with FEP and healthy controls.

	FEP (*N* = 142)		HCs (*N* = 142)		
Characteristic	Mean	SD	Mean	SD	*P*
Age (years)	25.2	7.6	26.4	8.1	0.077^a^
Gender (male/female)	61/81	-	61/81	-	1.000^b^
GAF	29.9	10.6	-	-	-
PANSS					
Total	89.7	16.9	-	-	-
Positive	24.9	6.4	-	-	-
Negative	19.1	8.3	-	-	-
General psychopathology	45.7	9.7	-	-	-

a *P* by Mann–Whitney test

b *P* by two-tailed Pearson chi-square test. Abbreviations: GAF, Global Assessment of Functioning Scale; *N*, number of participants; PANSS, Positive and Negative Syndrome Scale; SD, standard deviation.

**Table 4:** Significant changes in cortical thickness in patients with FEP compared to healthy controls in the independent sample.

	Talairach coordinates (peak)					
Brain region	*x*	*y*	*z*	*P*	Size (mm^2^)	Cluster breakdown
**Individuals with FEP < healthy controls**						
Right superior temporal cortex	40.1	1.9	20.9	0.0001	10 842	Superior temporal cortex
						Middle temporal cortex
						Insula
						Inferior temporal cortex
						Fusiform
Right superior frontal cortex	11.4	6.1	41.8	0.0001	4823	Superior frontal cortex
						Dorsal anterior cingulate cortex
						Posterior cingulate cortex
Left superior temporal cortex	−17.7	31.8	−59.3	0.0001	15 417	Superior temporal cortex
						Middle temporal cortex
						Insula
						Inferior temporal cortex
						Fusiform
Left superior frontal cortex	−10.6	0.7	41.8	0.0001	8992	Superior frontal cortex
						Dorsal anterior cingulate cortex
						Posterior cingulate cortex
						Precentral cortex
**Individuals with FEP > healthy controls**						
Right medial occipital cortex	10.2	−69.6	17.1	0.0001	5767	Lingual cortex
						Cuneus cortex
Right prefrontal cortex	9.4	52.5	−5.9	0.0001	5722	Prefrontal cortex
Left medial occipital cortex	−6.9	−80.6	5.2	0.0001	4704	Lingual gyrus
						Cuneus gyrus

## Discussion

We used a coordinate based meta-analysis approach to define the most robust cortical thickness alterations in patients with FEP, and to assess the effects of clinical and demographic factors. We found three regions within the default mode network and salience network that showed lower CTh (i.e. cortical thinning): the right middle temporal cortex extending to superior temporal cortex, insula, and anterior cingulate cortex. Previous meta-analyses have reported lower gray matter volume in the STC, insula, and ACC in FEP, which may be part of its neural substrate (Fusar-Poli *et al*., 2014; Radua *et al*., 2012a; Shah *et al*., 2017). There was no significant effect of age, gender, illness duration, or medication status on CTh in any of the three identified clusters.

Replication in an independent sample is a particular strength of this study. Consistent with the meta-analysis, the whole-brain analysis showed qualitatively similar abnormalities of clusters located in the MTC, STC, insula, and ACC. The ROI analysis also supported these results. Interestingly, we found no significant associations between clinical symptom severity and CTh, suggesting that cortical thinning may be a trait marker of predisposition to FEP rather than a manifestation of the disease (Brent *et al*., 2013). Participants with psychotic experiences or at genetic/clinical high risk for developing psychiatric disorders show a decline in gray matter in the temporal, frontal, and cingulate cortices, which persisted in those who transformed to psychosis (Merritt *et al*., 2021). Consistent with this, cortical thinning in the STC, ACC, and insula has been found in both psychiatric patients and their healthy first-degree relatives (Oertel-Knöchel *et al*., 2013), suggesting that these CTh reductions might represent an endophenotype of psychosis.

The most prominent cortical thinning in FEP was in the temporal cortex, especially STC and MTC. This region is located in the lateral portion of temporal cortex, and is a component of the DMN (Mulders *et al*., 2015). The DMN is crucial in thought processes, autobiographical memory, and conceiving others’ perspectives (Buckner *et al*., 2008), and dysfunction of the DMN, particularly the STC/MTC, is related to core symptoms of psychosis (Mallikarjun *et al*., 2018; O'Neill *et al*., 2019). The STC is thought to contain the auditory association cortical areas (Sun *et al*., 2009) and is a potential candidate for the neural basis of auditory hallucinations in psychosis (Allen *et al*., 2008). The STC also plays an important role in cognitive impairment and disorganized behavior in psychosis, which is related to loss of CTh (Kim *et al*., 2012; Walton *et al*., 2017). The MTC, particularly the middle part delineated in our study, plays an important role in semantic memory (Chang *et al*., 2011; Squire, 1992; Xu *et al*., 2015). Semantic memory dysfunction manifests as loss of general knowledge and information (Hui *et al*., 2012; Tan *et al*., 2020) increasingly considered important in psychosis because semantic memory deficits are related to the severity of core symptoms, including delusions (Rossell *et al*., 1999) and disorganized thought (Tan and Rossell, 2014).

We also found lower CTh in the insula and ACC, which are important hubs of the SN (Mulders *et al*., 2015). A review has identified the cardinal role of the SN in early-stage psychosis (Palaniyappan and Liddle, 2012b). The SN is essential in detection and integration of emotional and sensory stimuli (Downar *et al*., 2000). Misattribution of salience to external and internal stimuli has been proposed as a core feature of psychosis, underlying psychotic symptoms such as delusions and hallucinations (Palaniyappan and Liddle, 2012b). The insular cortex has extensive interconnectivity with many cortical areas and the limbic system (Jang *et al*., 2006). Insula dysfunction may lead to abnormalities in processes related to salience processing (Menon and Uddin, 2010), emotional appraisal, and social cognition (Eckert *et al*., 2009) that are characteristic of psychosis (Wylie and Tregellas, 2010). ROI analyses support insular cortical thinning in FEP (Roiz-Santianez *et al*., 2010; Wannan *et al*., 2019). Possible mechanisms include anomalies in cortical maturation, including cellular shrinkage, reduction in neuropil volume, and progressive changes in myelination (Roiz-Santianez *et al*., 2010).

The ACC is crucial in affective, executive, and cognitive functions (Fornito *et al*., 2009; Radua *et al*., 2012a). A meta-analysis of postmortem studies revealed lower density of nonpyramidal neurons in the ACC in psychotic individuals, which might form the cytological basis of cortical thinng (Todtenkopf *et al*., 2005). Dysfunction of ACC is well characterized for negative symptoms in psychosis, and ACC thinning might be involved in the increasing social withdrawal, which is a characteristic of the psychosis prodrome (Bersani *et al*., 2014). In support of this, FEP patients with persistent negative symptoms (PNS) showed a thinner cortex in the right ACC compared with HCs and FEP patients without PNS (Bodnar *et al*., 2014).

In subgroup analysis of studies done on 1.5 T scanners, results remained largely consistent with the pooled meta-analysis, with one additional significantly lower-CTh cluster in the right inferior frontal cortex and one higher-CTh cluster in the left precentral cortex. Since 8 out of 11 of the included datasets were at 1.5 T, this concordance is reasonable. The lack of similar results in the 3 T subgroup may due to the limited number of datasets (3/11 of the included datasets). The results of subgroup analyses should be regarded with caution: the minimum number of studies recommended for subgroup analyses in SDM software is 10 (Radua *et al*., 2014a). Future studies with larger samples and consistent field strength are needed to confirm this finding.

We further propose that the aforementioned brain regions do not affect the disease individually, but as an integration. For example, patients with FEP showed decreased integrity in the white matter fibers (such as cingulum) connecting the cingulate cortex and temporal areas (Sun *et al*., 2015), which may be a cause or a consequence of abnormalities in the gray matter (Konrad and Winterr, 2008). In terms of brain function, a recent meta-analysis comparing FEP with HCs reports hypo-connectivity between SN (insula and ACC) and DMN (particularly MTC), associated with perception anomalies (O'Neill *et al*., 2019). In addition, a multimodal meta-analysis has identified coupling changes between cortical structure (lower gray matter volume) and function (hyper-activation or hypo-activation) in these regions, and suggested a causal link: hyper-activation may cause gray matter loss by "exhaustion," while hypo-activation may cause the same as a compensation to avoid neuronal underemployment (Radua *et al*., 2012a). Our meta-analysis adds information on cortical thinning.

Interestingly, cortical thinning in our meta-analysis only presented on the right hemisphere, which is consistent with the theory of psychosis as a disturbance of lateralization (Crow *et al*., 2013). In fact, asymmetry is ubiquitous in the human brain (de Kovel *et al*., 2019). An older study suggested that auditory hallucinations, the core feature of psychosis, arise from the right hemisphere, and perhaps because the lack of characteristic of being self-generated (Nasrallah, 1985). The right hemisphere is important in mediating higher language functions, of which the deficits displayed by psychosis patients may make a significant contribution to their social interaction deficits (Mitchell and Crow, 2005). Other psychotic symptoms of FEP, such as lack of insight and suicidal behavior, were linked with unilateral gray matter reduction in the right hemisphere (Canal-Rivero *et al*., 2020; Tordesillas-Gutierrez *et al*., 2018). Moreover, factors such as handedness, sex, and disease processes associated with psychosis have been suggested to modulate the structural lateralization of the cerebral hemispheres (Hamilton *et al*., 2007). We deduced that the right hemisphere lateralization of cortical thickness thinning was a combined effect of multiple causes. Future studies that will clarify the specific contribution of the right hemisphere to psychopathological mechanisms are expected.

The present study, together with previous findings of structural and functional abnormalities in insula, ACC, and MTC in FEP (Cui *et al*., 2018; Palaniyappan and Liddle, 2012a; Radua *et al*., 2012a), strongly implicates the SN and DMN in the pathogenesis of FEP.

### Limitations

Our study has some limitations. A general limitation of coordinate-based meta-analysis is that using published coordinates instead of *t*-statistic brain maps limits accuracy (Radua *et al*., 2012b). Second, given the mixed clinical and imaging characteristics of our samples and the lack of some key information (e.g. diagnosis type, medication type and dosage, spatial resolution), we were unable to make much use of subgroup or regression analyses. Third, our study was restricted to cortical areas, and subcortical regions such as hippocampus, amygdala, and thalamus remain to be explored. Fourth, group-level inferences employing traditional mass-univariate neuroimaging approaches in our study restricted the information to make diagnostic decisions about FEP patients (Vieira *et al*., 2020).

## Conclusion

In summary, in a whole-brain meta-analysis in FEP, we found prominent cortical thinning involving the DMN (i.e. MTC and STC) and SN (i.e. insula and ACC), which was replicated in an independent sample of patients and control participants. We suggest that these reflect the neuropathologic basis of deficits of salience processing, and cognitive and emotional integration, which are important in FEP. Future studies with medication-naïve FEP participants will help verify our conclusions. Longitudinal studies will provide crucial information in understanding the clinical progress of CTh alterations from high psychosis risk to chronic status.

## Supplementary Material

kkab015_Supplemental_Files
