# Supplementary material for: Cortical thickness abnormalities in patients with first episode psychosis: a meta-analysis of psychoradiologic studies and replication in an independent sample
Source: Psychoradiology. 2021 Dec 15;1(4):185–98. doi: 10.1093/psyrad/kkab015 (PMC8826222; doi:10.1093/psyrad/kkab015)
Supplement: kkab015_Supplemental_Files [file kkab015_Supplemental_Files.zip › Supplementary Materials.docx]

Supplementary Materials

Contents

Supplementary Methods

Table S1. Newcastle-Ottawa scale assessment of risk of bias for case-control studies

Table S2. Summary of imaging characteristics and cortical thickness (CTh) alterations in 11 datasets from individuals with first-episode psychosis

Table S3. Results of the jackknife analysis of the meta-analysis

Table S4. Results of subgroup meta-analysis of studies at 1.5 T

Table S5. The findings of the meta-regression analyses

Table S6. Results of ROI-based analysis

Table S7. Results of correlation analyses between the extracted CTh values of each ROI region and demographic and clinical characteristics in the independent sample

Figure S1. Flowchart of literature search and selection criteria

Figure S2. Risk of bias summary table for each included study

Figure S3. Risk of bias of all included studies

Figure S4. Funnel plot for publication bias analysis: individuals with first episode psychosis compared with healthy controls

Figure S5. ROI masks extracted from the meta-analysis results of cortical thinning in first episode psychosis compared with healthy controls

# Supplementary Methods

## 1. Participants

One hundred and forty-two subjects with first episode psychosis were recruited from the Mental Health Centre of West China Hospital. All patients were antipsychotic-naïve and fulfilled the operational criteria for schizophrenia in the Structured Clinical Interview for DSM-IV (SCID) Patient Version. One hundred and forty-two age- and sex-matched healthy controls (HCs) were recruited by poster advertisements from the local area, and SCID Non-patient Edition was used to conﬁrm the lifetime absence of psychiatric and neurological illness in both HCs and their ﬁrst-degree relatives. Exclusion criteria for both groups included: (1) aged under 18 or over 60 years, (2) history of traumatic head injury, (3) history of alcohol or substance abuse, (4) history of neurologic illness, (5) history of serious medical or surgical illness, (5) current pregnancy, and (6) claustrophobia or other contraindications to MRI examination, as assessed by two experienced psychiatrists in a detailed clinical interview.

## 2. MRI date acquisition

All subjects were scanned in a 3.0 T magnetic resonance scanner (EXCITE, General Electric, Milwaukee) with an 8-channel head coil. The T1-weighted images were obtained using 3D spoiled gradient echo sequence with the following acquisition parameters: TR 8.5 ms; TE 3.4 ms; ﬂip angle 12°; 156 contiguous coronal slices with thickness of 1.0 mm; FOV 240 × 240 mm^2^; data matrix 256 × 256; voxel size 0.47 × 0.47 × 1 mm^3^. The participants were laid supine comfortably on the moving couch. Foam pads were used to stabilize the head and soft earplugs were used to reduce noise. During scanning, the subjects were instructed to keep their eyes closed, neither fall asleep nor think of anything in particular, and keep as still as possible.

# Table S1. Modified Newcastle-Ottawa scale assessment of risk of bias for case-control studies

Note: A study can be awarded a maximum of one star (*) for each numbered item within the Selection category. A maximum of two stars can be given for Comparability. If the item is awarded one star (*), it is considered at low risk. If the item is awarded no star, it is considered at high risk.

Selection

1) Is the case definition adequate?

a) Yes, with independent validation*

b) Yes, eg record linkage or based on self-reports

c) No description

2) Representativeness of the cases

a) Consecutive or obviously representative series of cases*

b) Potential for selection biases or not stated

3) Selection of Controls

a) Community controls*

b) Hospital controls

c) No description

4) Definition of Controls

a) No history of disease (endpoint) *

b) No description of source

Comparability

1) Comparability of cases and controls on the basis of the design or analysis

a) Study controls for “age”.*

b) Study controls for “gender”.*

# Table S2. Summary of imaging characteristics and cortical thickness (CTh) alterations in 11 datasets from individuals with first-episode psychosis

| Paper | MRI scanner | Field (T) | Sequence | TR/TE (ms) | Spatial resolution (mm^3^) | Software | Normalization template | Analytic model | Correction method |
| --- | --- | --- | --- | --- | --- | --- | --- | --- | --- |
| Ansell et al., 2015  FGA | GE Signa | 1.5 | SPGR | 14.3/3.3 | 0.94 × 0.94 × 1.5 | FreeSurfer | MNI | Vertex-based | P < 0.05 (MCS) |
| Ansell et al., 2015  SGA | GE Signa | 1.5 | SPGR | 14.3/3.3 | 0.94 × 0.94 × 1.5 | FreeSurfer | MNI | Vertex-based | P < 0.05 (MCS) |
| Buchy et al., 2018 | Siemens Magnetom Sonata | 1.5 | GRE | 22/9.2 | NA × NA × 1 | CIVET | MNI | Vertex-based | P < 0.05 (RFT) |
| Dukart et al., 2017 | Siemens Magnetom Verio | 3 | MPRAGE | 2000/3.4 | 1 × 1 × 1 | SPM12 | MNI | Voxel-based | P < 0.05 (PT) |
| Gutierrez et al., 2010 | GE Signa | 1.5 | IR SPGR | 15/5.4 | 1.2 × 1.2 ×1.2 | FreeSurfer | MNI | Vertex-based | P < 0.05 (FDR) |
| Haukvik et al., 2016 | Siemens Magnetom Sonata | 1.5 | MPRAGE | 2730/3.93 | 1.33 × 0.94 ×1 | FreeSurfer | Tal | Vertex-based | P < 0.05 (FDR) |
| Lesh et al., 2015 | GE Signa | 1.5 | SPGR | 9/2 | 0.86 × 0.86 × 1.5 | FreeSurfer | Tal | Vertex-based | P < 0.05 (MCS) |
| Lin, et al., 2019 | GE Signa | 3 | SPGR | 8.5/3.93 | 0.47 × 0.47 × 1 | FreeSurfer | Tal | Vertex-based | P < 0.05 (FDR) |
| Rais et al., 2010 | Philips NT | 1.5 | FFE | 30/4.6 | NA × NA × 1.2 | CLASP | Tal | Vertex-based | P < 0.10 (FDR, 1-tail) |
| Reniers et al., 2015 | Siemens Magnetom Trio | 3 | NA | 3.6/9 | 1.0 × 0.5 × 0.5 | FreeSurfer | MNI | Vertex-based | P < 0.05 (MCS) |
| Scalon et al., 2014 | Siemens Magnetom Symphony | 1.5 | MPRAGE | 1140/4.38 | 0.9 × 0.9 × 0.9 | FreeSurfer | MNI | Vertex-based | P < 0.05 (FDR) |
| Abbreviations: FFE, fast field echo; FGA, first generation antipsychotics; FDR, false discovery rate; FWE, Family-Wise Error; GRE, gradient echo; HC, healthy controls; IR, inversion recovery; MCS, Monte Carlo simulations; MNI, Montreal Neurological Institute; NA, not applicable; PT, permutation test; RFT, Random ﬁeld theory; SGA, second generation antipsychotics; T, tesla; Tal, Talairach; TE, echo time; TR, repetition time; MPRAGE, magnetization prepared rapid gradient echo. SPGR, spoiled gradient recalled echo. | | | | | | | | | |

# Table S3. Results of the jackknife analysis of the meta-analysis

| Discarded study | Right middle temporal cortex | Right insula | Right anterior cingulate cortex |
| --- | --- | --- | --- |
| Ansell et al., 2015 FGA | Yes | Yes | Yes |
| Ansell et al., 2015 SGA | Yes | No | No |
| Buchy et al., 2018 | Yes | Yes | Yes |
| Dukart et al., 2017 | Yes | Yes | Yes |
| Gutierrez et al., 2010 | Yes | Yes | Yes |
| Haukvik et al., 2016 | Yes | Yes | Yes |
| Lesh et al., 2015 | Yes | Yes | Yes |
| Lin, et al., 2019 | Yes | Yes | Yes |
| Rais et al., 2010 | Yes | Yes | Yes |
| Reniers et al., 2015 | Yes | Yes | Yes |
| Scalon et al., 2014 | No | Yes | Yes |
| Total | 10/11 | 10/11 | 10/11 |

# Table S4. Results of subgroup meta-analysis of studies at 1.5 T

| Brain regions | MNI coordinate | | | SDM | P, | Voxels | Cluster breakdown (voxels, n) |
| --- | --- | --- | --- | --- | --- | --- | --- |
|  | x | y | z | (z score) | uncorrected | (n) |  |
| Individuals with first episode psychosis > healthy controls | | | | | | | |
| Left precentral cortex | -36 | -18 | 46 | 1.038 | 0.000013 | 403 | Left precentral cortex, BA 3, 4, 6 (145) |
|  |  |  |  |  |  |  | Left precentral cortex, BA 3, 4, 6 (258) |
| Individuals with first episode psychosis < healthy controls | | | | | | | |
| Right middle temporal cortex | 44 | -30 | -8 | -1.072 | 0.00192 | 298 | Right middle temporal cortex, BA 20, 21, 22, 48 (181) |
|  |  |  |  |  |  |  | Right superior temporal cortex, BA 21, 22, 48 (117) |
| Right insular cortex | 40 | 2 | 14 | -1.197 | 0.00079 | 185 | Right insula cortex, BA 48 (119) |
|  |  |  |  |  |  |  | Right Rolandic operculum, BA 48 (66) |
| Right anterior cingulate cortex | 14 | 32 | 28 | -1.076 | 0.0019 | 45 | Right anterior cingulate/paracingulate cortex, BA 32 (45) |
| Right inferior frontal cortex | 42 | 32 | 26 | -1.065 | 0.0021 | 42 | Right inferior frontal cortex, BA 45, 46 (27) |
|  |  |  |  |  |  |  | Right middle frontal cortex BA 45, 46 (15) |
| Abbreviations: BA, Brodmann area; MNI, Montreal Neurological Institute; SDM, seed-based d mapping. | | | | | | | |

| Meta-regression | Right middle temporal cortex | | Right insular cortex | | Right anterior cingulate cortex | |
| --- | --- | --- | --- | --- | --- | --- |
|  | P | r | P | r | P | r |
| Age | 0.122 | -0.494 | 0.436 | 0.262 | 0.436 | 0.262 |
| Female percentage | 0.938 | -0.027 | 0.715 | -0.124 | 0.715 | -0.124 |
| Illness duration | 0.631 | 0.174 | 0.625 | 0.177 | 0.625 | 0.177 |
| Medication percentage | 0.628 | -0.175 | -0.308 | 0.368 | -0.308 | 0.368 |

# Table S5. The findings of the meta-regression analyses

# Table S6. Results of region-of-interest-based analysis

| Brain regions | First episode psychosis | | Healthy controls | | P† |
| --- | --- | --- | --- | --- | --- |
|  | Mean | SD | Mean | SD |  |
| Right middle temporal cortex | 2.55 | 0.16 | 2.69 | 0.17 | <0.0001 |
| Right insular cortex | 2.39 | 0.16 | 2.62 | 0.19 | <0.0001 |
| Right anterior cingulate cortex | 2.49 | 0.31 | 2.79 | 0.27 | <0.0001 |
| †P by two-sample t-test with false discovery rate correction. | | | | | |

# Table S7. Results of the correlation analyses between the extracted CTh values of each ROI region and demographic and clinical characteristics in the independent sample

| Correlation analysis | Right middle temporal cortex | | Right insular cortex | | Right anterior cingulate cortex | |
| --- | --- | --- | --- | --- | --- | --- |
|  | P | r | P | r | P | r |
| Age | **0.18** | **-0.19** | **0.32** | **-0.15** | **0.56** | **-0.10** |
| GAF | **0.97** | **-0.004** | **0.59** | **0.08** | **0.97** | **0.03** |
| PANSS total | *0.76* | *0.05* | *0.76* | *-0.03* | *0.76* | *-0.04* |
| PANSS positive | *0.72* | *-0.12* | *0.76* | *-0.06* | *0.72* | *-0.12* |
| PANSS negative | **0.56** | **0.10** | **0.97** | **-0.003** | **0.97** | **0.01** |
| PANSS general | *0.76* | *0.09* | *0.76* | *-0.04* | *0.76* | *-0.03* |
| Italicized R- and p-values are Spearman rank correlation with false discovery rate correction, and the remaining R and P values in **bold** are Pearson correlation with false discovery rate correction.  Abbreviations: GAF, Global Assessment of Functioning scale; PANSS, Positive and Negative Syndrome Scale. | | | | | | |

Figure S1. Flowchart of literature search and selection criteria.


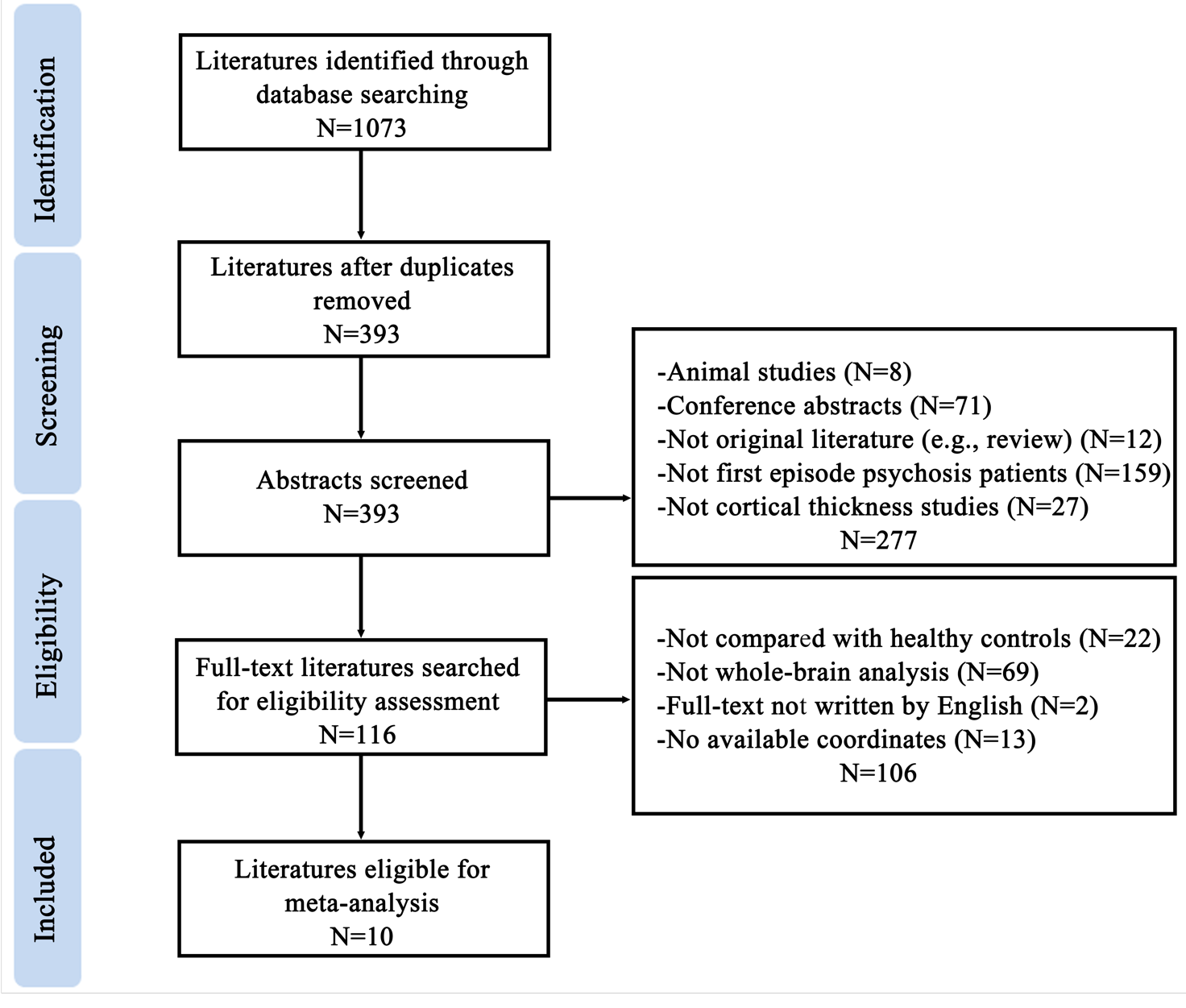


Figure S2. Risk of bias summary table for each included study
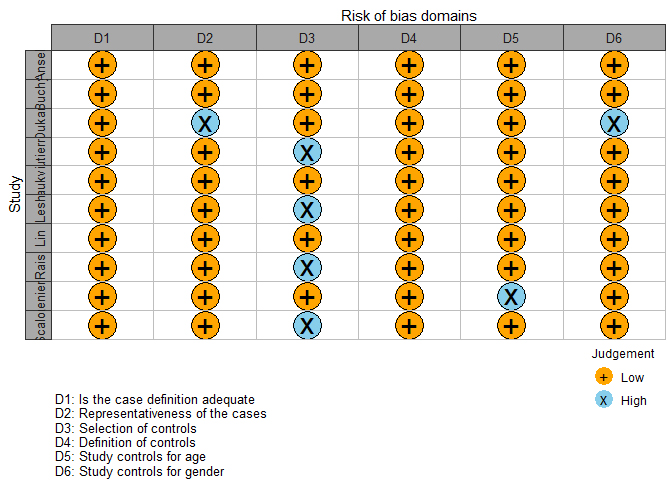


# Figure S3. Risk of bias of all included studies


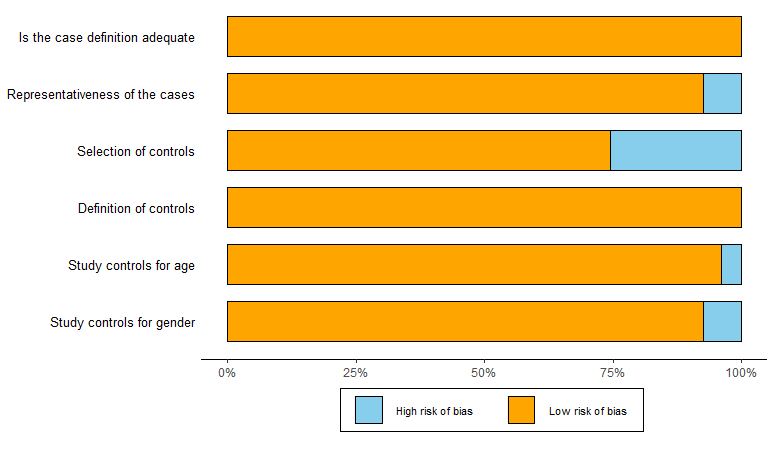


Figure S4. Funnel plot analysis for publication bias: individuals with first episode psychosis compared with healthy controls


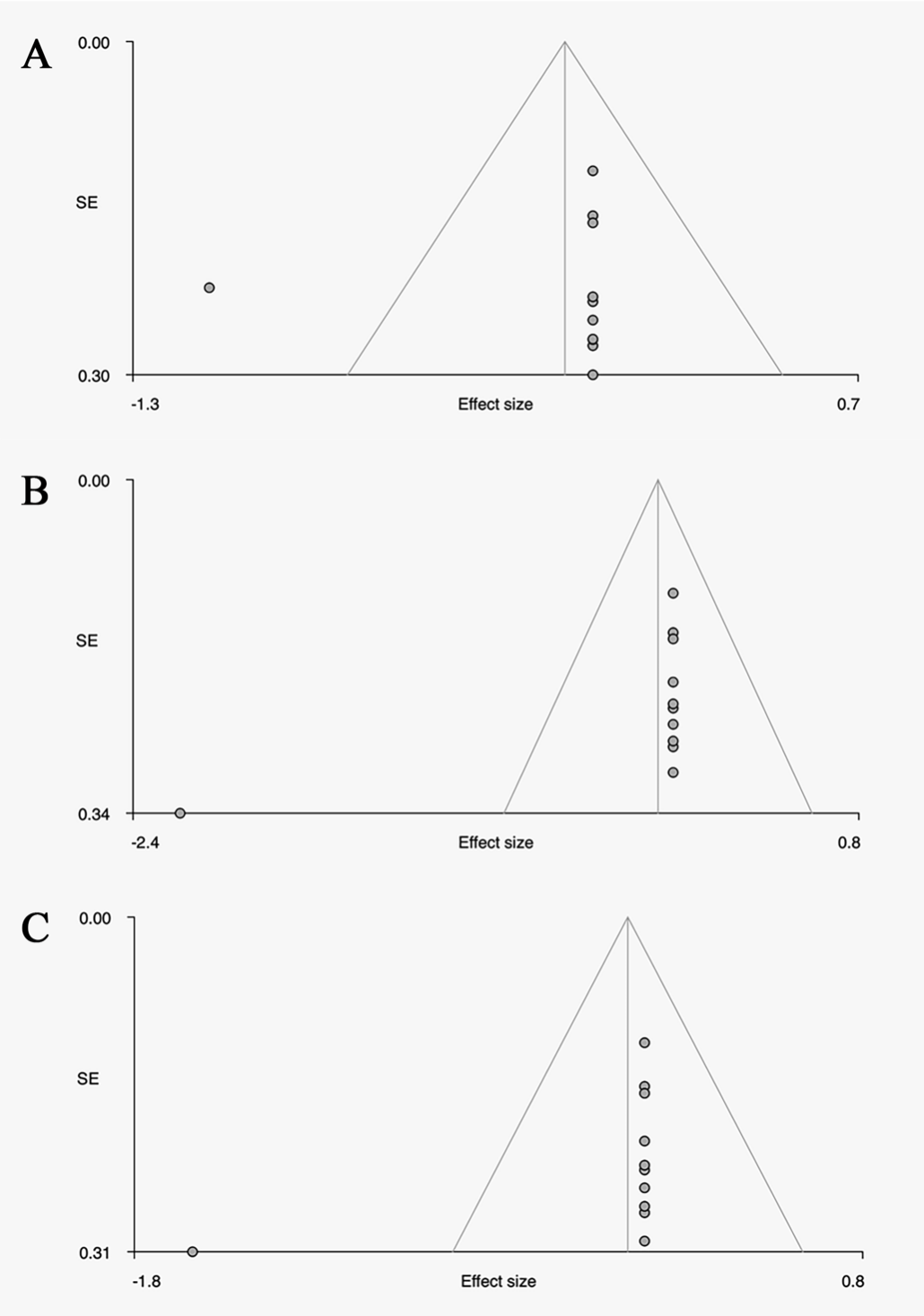


Egger’s test and funnel plots revealed no significant publication bias (A) in the right middle temporal gyrus (z = -0.67, t = -0.45, df = 9, p = 0.662), (B) insula (z = -2.51, t = -1.43, df = 9, p = 0.188), and (C) anterior cingulate cortex (z = -1.85, t = -1.25, df = 10, p = 0.243).

# Figure S5. ROI masks extracted from the meta-analysis results of cortical thinning in first episode psychosis compared with healthy controls

Abbreviations: ACC, anterior cingulate cortex; MTC; middle temporal cortex.
