# Supplementary material for: Cortical thickness abnormalities in patients with first episode psychosis: a meta-analysis of psychoradiologic studies and replication in an independent sample
Source: Psychoradiology. 2021 Dec 15;1(4):185–98. doi: 10.1093/psyrad/kkab015 (PMC8826222; doi:10.1093/psyrad/kkab015)
Supplement: kkab015_Supplemental_Files [file kkab015_Supplemental_Files.zip › Response to the reviewers.docx]

Response to the reviewers

We would like to thank the editor and reviewer for their encouraging and helpful comments. In our revision, changes have been marked in **b****lue.** Detailed responses to these comments are summarized as follows.

***Reviewer 1:***

*It would be useful to see one table which summarises results by other studies according to the results, in particular, CTh in MTC, STC, insula, ACC as well as demographic and clinical variables, by this meta-analysis. I think you can take information from Table S2 (main findings) and S3, and remake one summary table.*

**Response**：Thanks a lot for the reviewer’s suggestion. We have now remade the Table 1to contain both demographic and clinical variables and main findings of each included study. However, Table S3 shows the results of jackknife sensitivity analysis of our meta-analysis rather than CTh alterations in each included study, so we did not emerge it into Table 1.

***Reviewer 2:***

1. *Abstract: Abstract is clear enough to give an idea of the manuscript content, however I would suggest to rephrase the expression "the most prominent CTh changes". The meta- analysis indicates consist and general results, but not necessarily the most prominent.*

**Response:** We thank for the reviewer’s comments. We agree that the meta-analysis indicates consist and general results, but not necessarily the most prominent. We have revised the sentence in the abstract (page 2, paragraph 2, line 1) as follows:

“the most consistent CTh changes”.

2. *Introduction: In line 20-22 of page 3, the SBM method may be not a recent methodological advance.*

**Response:** Thanks for the reviewer’s comments. Following the reviewer’s suggestions, we have revised the sentence (page 3, paragraph 1, line 7) as follows:

“Applying surface-based morphometric methods allows quantification of cortical thickness (CTh) (Fischl et al., 2000)”.

3. *Material and Methods: In line 10-17 of page 5, why did the search terms include "drug-naïve bipolar disorder" and "drug-naïve depression"? As you said, FEP was defined as several mental illnesses characterized by psychotic symptoms including schizophrenia spectrum psychoses (schizophrenia, schizoaffective, schizophreniform) and affective psychoses (bipolar psychosis and psychotic depression). If you do that, more studies can be included into the meta-analysis to obtain more robust results.*

**Response:** Thanks for this great suggestion. We have added several terms (*“first episode bipolar disorder”, “early bipolar disorder”, “drug-naïve bipolar disorder”, “first episode depression”, “early depression”, and “drug-naïve depression”*) to search for further inclusion in the *Web of Science*, *PubMed*, *Embase* and *Science Direct* databases. After removing the 123 duplicates, 110 more studies were identified. One hundred out of 110 studies were excluded due to the following reasons: not at the first episode of disease (N = 58), not whole-brain analysis (19), not compared with healthy controls (N = 5), animal studies (N = 5), not cortical thickness studies (N = 3), review (N = 3), and conference abstracts (N = 2). The remaining 10 studies that recruited first episode depression patients were excluded from our meta-analysis as well, because these first episode depression patients lacked psychotic symptoms, such as hallucinations and delusions, that could not be diagnosed as first episode psychosis (Lieberman et al., 2018). And we have updated the *Material and Methods* and *Figure S1* accordingly.

4. *Material and Methods: In Statistical Analysis, before the correlation analyzes and two-sample t-tests, please check whether the variables (CTh values of vertexes and ROIs) obey normal distribution.*

**Response:** We thank for the reviewer’s suggestions. We checked whether the clinical variables and CTh values of each ROI region were normally distributed using Kolmogorov-Smirnov (K-S) tests, Shapiro-Wilk tests and visual assessment of the histogram (taking statistical significance as P < 0.05) (Ghasemi et al., 2012). Normally distributed parameters included PANSS total, positive and general scores and CTh values of all three ROIs. Non-normally distributed parameters included age, GAF scores, PANSS negative scores. Comparisons between groups was performed with two-sample Student’s t-tests for normally distributed variables and the Mann–Whitney rank-sum tests for non-normal data. Pearson χ2 tests were used to compare gender distribution between groups. Correlations were calculated with Pearson correlations for normally distributed clinical parameters and Spearman correlations for non-normally distributed parameters.

Statistical results showed that FEP patients and HCs did not significantly differ in age and gender, FEP patients showed significantly lower CTh in all three ROIs, and there was no significant association between CTh and clinical characteristics, which were consistent with our previous results. And we have updated the *Statistical Analysis, Table 3 and Table S7* accordingly.

For whole-brain vertex-wise analysis, a general linear model (GLM) based on QDEC (Query, Design, Estimate, Contrast) of FreeSurfer was used. This model assumes that the data will follow a normal distribution. Indeed, smoothing compensates for residual anatomical variability after spatial normalization and renders the CTh more normally distributed increasing the validity of any statistical parametric test (Hutton et al., 2008). The GLM method in the analysis of cortical thickness is widely applied and has proven reliable (Cardinale et al., 2014). Given that the necessity of normality tests in vertex-based whole-brain analysis is proofless, we adopted vertex-wise comparison using GLM without normality tests.

5. *Results: In the Results of the pooled meta-analysis, how do you explain CTh differences only showing in the right hemisphere?*

**Response:** We thank for the reviewer’s comments. We have supplemented the possible explanation that why CTh differences were only shown in the right hemisphere in the Discussion (page 15, paragraph 2) as follows:

“Interestingly, cortical thinning in our meta-analysis only presented on the right hemisphere, which is consistent with the theory of psychosis as a disturbance of lateralization (Crow et al., 2013). In fact, asymmetry is ubiquitous in the human brain (de Kovel et al., 2019). An older study suggested that auditory hallucinations, the core feature of psychosis, arise from the right hemisphere, and perhaps because the lack of characteristic of being self-generated (Nasrallah 1985). The right hemisphere is important in mediating higher language functions, of which the deficits displayed by psychosis patients may make a significant contribution to their social interaction deficits (Mitchell et al., 2005). Other psychotic symptoms of FEP, such as lack of insight and suicidal behavior, were linked with unilateral gray matter reduction in the right hemisphere (Canal-Rivero et al., 2020; Tordesillas-Gutierrez et al., 2018). Moreover, factors such as handedness, sex, and disease processes associated with psychosis have been suggested to modulate the structural lateralization of the cerebral hemispheres (Hamilton et al., 2007). We deduced that the right hemisphere lateralization of cortical thickness thinning was a combined effect of multiple causes. Future research that will clarify the specific contribution of right hemisphere to psychopathological mechanisms is expected”.

6. *In the section of Subgroup meta-analysis, how do you explain the results that the significant findings were replicated in your own database using 3.0 T scanner, but not the 3 datasets using 3.0 T scanner in previous studies?*

**Response:** Thanks a lot for the reviewer’s comment. Despite the consistent field strength of our own dataset and the 3.0 T subgroup, the sample size and demographic and clinical characteristics of the above analyses are inconsistent, which may contribute to the different results. The inconsistency between the 3.0 T subgroup analysis and pooled meta-analysis may be due to the limited number of datasets (3/11 of the included datasets).

7. *As you said in the Introduction section, abundance of structural magnetic resonance imaging studies provided key information for neurostructural changes in FEP (Li et al., 2021). How about the CTh differences from the meta-analysis? Can they provide any reference for FEP diagnosis and be regarded as imaging markers in your own database?*

**Response:** Thanks for the reviewer’s comments. The meta-analysis aimed to synthesize the multitude of results from different studies in an unbiased way and to identify consistent results. It may offer insights that are not immediately apparent from the individual studies (Quah et al., 2017). However, these neuroimaging studies typically make group-level inferences, rendering it difficult to assess individual patients or develop personalized diagnoses. Future studies that employ machine learning methods may provide predictive models to make inferences at the individual diagnosis (Vieira et al., 2020). And we have addressed this to the Limitation of the study (page 16, paragraph 3, line 7) as follows:

“Fourth, group-level inferences employing traditional mass-univariate neuroimaging approaches in our study restricted the information to make diagnostic decisions about FEP patients (Vieira et al., 2020)”.

**References**

1. Canal-Rivero, M., Tordesillas-Gutiérrez, D., Ruiz-Veguilla, M.et al. (2020) Brain grey matter abnormalities in first episode non-affective psychosis patients with suicidal behaviours: The role of neurocognitive functioning. *Prog Neuropsychopharmacol Biol Psychiatry.* 102:109948.
2. Cardinale, F., Chinnici, G., Bramerio, M.et al. (2014) Validation of FreeSurfer-estimated brain cortical thickness: comparison with histologic measurements. *Neuroinformatics.* 12 (4):535-42.
3. Crow, T. J., Chance, S. A., Priddle, T. H.et al. (2013) Laterality interacts with sex across the schizophrenia/bipolarity continuum: an interpretation of meta-analyses of structural MRI. *Psychiatry Res.* 210 (3):1232-44.
4. de Kovel, C. G. F., Aftanas, L., Aleman, A.et al. (2019) No Alterations of Brain Structural Asymmetry in Major Depressive Disorder: An ENIGMA Consortium Analysis. *Am J Psychiatry.* 176 (12):1039-1049.
5. Fischl, B., and Dale, A. M. (2000) Measuring the thickness of the human cerebral cortex from magnetic resonance images. *Proc Natl Acad Sci U S A.* 97 (20):11050-5.
6. Ghasemi, A., and Zahediasl, S. (2012) Normality tests for statistical analysis: a guide for non-statisticians. *Int J Endocrinol Metab.* 10 (2):486-9.
7. Hamilton, L. S., Narr, K. L., Luders, E.et al. (2007) Asymmetries of cortical thickness: effects of handedness, sex, and schizophrenia. *Neuroreport.* 18 (14):1427-31.
8. Hutton, C., De Vita, E., Ashburner, J.et al. (2008) Voxel-based cortical thickness measurements in MRI. *Neuroimage.* 40 (4):1701-10.
9. Lieberman, J. A., and First, M. B. (2018) Psychotic Disorders. *N Engl J Med.* 379 (3):270-280.
10. Mitchell, R. L., and Crow, T. J. (2005) Right hemisphere language functions and schizophrenia: the forgotten hemisphere? *Brain.* 128 (Pt 5):963-78.
11. Nasrallah, H. A. (1985) The unintegrated right cerebral hemispheric consciousness as alien intruder: a possible mechanism for Schneiderian delusions in schizophrenia. *Compr Psychiatry.* 26 (3):273-82.
12. Quah, Stella R, and Cockerham, William C eds.2017 International encyclopedia of public health, 2nd edition: Academic Press.
13. Tordesillas-Gutierrez, D., Ayesa-Arriola, R., Delgado-Alvarado, M.et al. (2018) The right occipital lobe and poor insight in first-episode psychosis. *PLoS One.* 13 (6):e0197715.
14. Vieira, S., Gong, Q. Y., Pinaya, W. H. L.et al. (2020) Using Machine Learning and Structural Neuroimaging to Detect First Episode Psychosis: Reconsidering the Evidence. *Schizophr Bull.* 46 (1):17-26.
